# Supplementary material for: Medication-related osteonecrosis of the jaws after tooth extraction in senescent female mice treated with zoledronic acid: Microtomographic, histological and immunohistochemical characterization
Source: PLoS One. 2019 Jun 14;14(6):e0214173. doi: 10.1371/journal.pone.0214173 (PMC6568384; doi:10.1371/journal.pone.0214173)
Supplement: S1 Table — (DOC) [file pone.0214173.s001.doc]

| **Supplementary Table 1. Summary of MRONJ mice models** | | | | | | |
| --- | --- | --- | --- | --- | --- | --- |
| **Author, year** | **Mice strain, age and sex** | **Drugs and dosages** | **Triggering Factor (TF)** | **Period of drug administration** | | **Experimental Periods** |
|  |  |  |  | **Before TF** | **After TF** |  |
| Kim T, Kim S et al, 2018 | C57BL/6, 6 wk-old, ♀ | Zoledronic acid (Zometa, Novartis Oncology) 125 µg/kg IV- 1x/2wk | Maxillary first molar extraction associated or not with PD (tooth ligature) | 1 wk before periodontal ligature 3 wk before TE | 3 wk after TE | 3 wk after TE |
|  |  |  |  |  |  |  |
|  |  | RANKL monoclonal Ab (IK22-5) 250 µg IV- 1x/2weeks |  |  |  |  |
| Kuroshima S et al, 2018 | C57BL/6J, 8-12 wk-old, ♀ | Zoledronate (Zometa, Novartis) 50 µg/kg SC- 2x/wk | TE of maxillary first molars | 3 wk | 4 wk | 4 wk |
|  |  | Cyclophosphamide (C7397; Sigma-Aldrich) 150 mg/kg IP- 2x/wk (before TE) and 1x/wk (after TE) |  |  |  |  |
|  |  |  |  |  |  |  |
| Akita Y et al, 2018 | C57BL/6J, 9 wk-old, ♂ | Zoledronate (Zometa, Novartis) 50 µg/kg SC- 2x/wk | TE of maxillary first molars | 3 wk | 4 wk | 2 and 4 wk |
|  |  | Cyclophosphamide (C7397; Sigma-Aldrich) 150 mg/kg IP- 2x/wk (before TE) and 1x/wk (after TE) |  |  |  |  |
|  |  |  |  |  |  |  |
| Kim S et al, 2017 | C57BL/6J and IL-1Rrp2^KO^, 8 wk-old, ♀ | Zoledronic acid (Zometa, Novartis Oncology) 125 µg/kg IV- 1x/2wk | TE of maxillary first molar | 1 wk | 3 wk (IL-1Rrp2^KO^) and 2 wk (others groups) | 2 and 3 wk |
|  |  |  |  |  |  |  |
| Molon RS, Hsu C et al, 2016 | DBA1/J, 10 wk-old, ♂ | Zoledronic Acid (Z-5744 LKT Laboratories) 200 μg/kg IP- 2x/wk | PD (drilling maxillary molars crown) | 1 wk | 8 wk | 8 wk |
|  |  |  |  |  |  |  |
| Movila A et al, 2016 | C57BL/6j, γδTcell^KO^, NR age and sex | Pamidronate disodium (LKT Laboratories) 2 mg/kg SC- 1x/wk | TE of maxillary first molar | 4 wk | NR | 15 d |
|  |  | Zoledronate (LKT Laboratories) 200 μg/kg SC- 1x/wk |  |  |  |  |
| Matsuura Y et al, 2016 | C57BL/6N, GFP-transgenic C57BL/6 N, 6 wk-old, ♂ | Zoledronate (Zometa, Novartis Oncology) 125 µg/kg IV- 2x/wk | TE of maxillary first molar | 1 wk | 1 wk | 1 wk |
|  |  |  |  |  |  |  |
|  |  | Dexamethasone (Sigma-Aldrich) 10 mg/kg IP- 2x/wk |  |  |  |  |
| Sun Y et al, 2016 | C57Bl/6J, 7 wk-old, ♀ | Zoledronate (Zometa; Novartis Oncology) 500 µg/kg IV- Single dose | TE of left maxillary first molar | 10 d | none | 3 d, 2 wk and 4 wk |
|  |  |  |  |  |  |  |
| Córdova LA et al, 2016 | C57BL/6J, 10 wk-old, ♂ | Zoledronic Acid (Novartis, Switzerland) 100 µg/kg IP- 2x/wk | TE maxillary first molar | 3 wk | 3 wk and 6 wk | 3 and 6 wk after TE |
|  |  |  |  |  |  |  |
| Sondia A et al, 2016 | C57BL/6J, 16 wk-old, ♂ | Zoledronic Acid (Z-5744 LKT Laboratories) 200 μg/kg IP- 2x/wk | Zoledronate (spontaneous periradicular disease) and TE | 8 wk | none | 4 wk after TE |
|  |  | OPG-Fc (Amgen, Inc) 10 mg/kg IP- 2x/wk |  |  |  |  |
| Song M et al, 2016 | C57BL/6, 6 wk-old, ♀ | Zoledronic acid (Zometa, Novartis Oncology) 125 µg/kg IV- 1x/2wk | PD induced for 3 wk and them TE (maxillary first molar) | 1 wk (for pulp exposure) 3 wk (for TE) | 6 wk (for pulp exposure) and 3 wk (TE) | 3 wk after TE |
|  |  |  |  |  |  |  |
|  |  |  |  |  |  |  |
| Zhang Q et al, 2015 | BKS.Cg-Dock7ᵐ+/+Leprᵈᵇ/J mouse (db/db), nondiabetic db/+ and B6.129S6-*Nlrp3*ᵗᵐ¹ʰᵏ/J, 6-8 wk-old, ♂ | Zoledronate (Novartis Pharma AG) 125 µg/kg IV- 2x/wk | TE maxillary first molars | 1 wk | 2 and 4 wk | 2 and 4 wk |
|  |  |  |  |  |  |  |
|  |  |  |  |  |  |  |
| [Park S et al, 2015](https://www.ncbi.nlm.nih.gov/pubmed/?term=Park S%5BAuthor%5D&cauthor=true&cauthor_uid=26013832) | C57Bl/6J, 7 wk-old, female | Zoledronate (Zometa; Novartis Oncology) 540 µg/kg IV- Single dose | TE maxillary first molar | 1 wk | none | 4 d, 1 wk, 2 wk and 4 wk |
|  | B6.129P2-Tcrdᵗᵐ¹ᴹᵒᵐ/J, 7 wk-old, ♀ |  |  |  |  |  |
|  |  |  |  |  |  |  |
| Su J et al, 2015 | C57BL/6J, 8-10 wk-old, ♀ | Zoledronate (Zometa, Novartis Oncology) 125 µg/kg IV- 2x/wk | TE maxillary first molar | 1 wk | 1 d, 1 wk, 3 wk and 8 wk | 1 d, 1 wk, 3 wk and 8 wk |
|  |  |  |  |  |  |  |
|  |  | Dexamethasone (Sigma-Aldrich) 5 mg/kg IP- 2x/wk |  |  |  |  |
| Molon RS, Shinamoto S et al, 2015 | C57BL/6J, 10 wk-old, ♂ | Zoledronic Acid (Z-5744 LKT Laboratories) 200 μg/kg IP- 2x/wk | PD (drilling mandibular molars crown) | 3 wk | 8 wk | 8 ,14 and 18 wk after drill |
|  |  | OPG-Fc (Amgen, Inc) 10 mg/kg IP- 2x/wk |  |  |  |  |
| Aghaloo TL et al, 2014 | C57BL/6J, 16 wk‐old, ♂ | RANK‐Fc (Amgen, Inc) 10 mg/kg IP- 3x/wk | PD (drilling maxillary molars crown) | 3 wk | 9 wk | 9 wk |
|  |  | OPG‐Fc (Amgen, Inc) 10 mg/kg IP- 1x/wk |  |  |  |  |
| Molon RS et al, 2014 | C57BL/6J, 16 wk-old, ♂ | Zoledronic Acid (Z-5744 LKT Laboratories) 200 μg/kg IP- 3x/wk | PD (drilling maxillary molars crown) | 3 wk | 9 wk | 9 wk |
|  |  | RANK-Fc (Amgen, Inc) 10 mg/kg IP- 3x/wk |  | 3 wk | 9 wk |  |
|  |  | OPG-Fc (Amgen, Inc) 10 mg/kg IP- 1x/wk |  | 3 wk | 9 wk |  |
| Williams DW et al, 2014 | C57BL/6, 8 wk-old, ♀ | RANKL monoclonal Ab (IK22-5) 250 µg IV- 1x/2wk | TE maxillary first molar | 1 wk | 3 wk | 4 wk |
|  |  | Zoledronic acid (Zometa, Novartis Oncology) 125 µg/kg IV- 1x/2wk |  | 1 wk | 3 wk |  |
| Zhang Q et al, 2013 | C57BL/6J, 8-10 wk-old, female; Beige nude/nude Xid (III), 8–10 wk-old, ♀ | Zoledronate (Zometa, Novartis Oncology) 125 µg/kg IV- 2x/wk | TE of maxillary first molar | 1 wk | 2 wk | 2 wk |
|  |  |  |  |  |  |  |
| Kang B et al, 2013 | C57BL/6J, 16 wk-old, ♂ | Zoledronic Acid (Z-5744 LKT Laboratories) 200 μg/kg IP- 3x/wk | PE (drilling mandibular molars crown) | 1 wk | 7 wk | 7 wk |
|  |  |  |  |  |  |  |
| Zhao Y et al, 2012 | C57BL/6J, 8 wk-old, ♀ | Zoledronate (Zometa, Novartis Oncology) 125 µg/kg IV- 2x/wk | TE of maxillary first molars | 1 wk | 2 wk | 2 wk |
|  |  |  |  |  |  |  |
|  |  | Dexamethasone (Sigma-Aldrich) 5 mg/kg IP- 2x/wk |  |  |  |  |
| Mawardi H, 2011 | C57BL/6j, 6-8 wk-old, ♀ | Pamidronate disodium (LKT Laboratories) 1 mg/kg SC- 1x/wk | TE maxillary first molars | 4 wk | none | 15 d |
|  |  |  |  |  |  |  |
| Bi Y et al, 2010 | C57BL6, 8-12 wk-old, NR sex | Zoledronate (NR) 125 µg/kg IP- 2x/wk | TE left maxillary or right mandibular first molar | 3 wk | 3 and 12 wk | 3 and 12 wk |
|  |  | Dexamethasone (NR) 5 mg/kg IP- 1x/wk |  |  |  |  |
|  |  | Docetaxel 25 mg/kg IP- 1x/wk |  |  |  |  |
| Kikuiri T et al, 2010 | C57BL/6J, 8-10 wk-old, ♀ | Zoledronate (Zometa, Novartis Oncology) 125 µg/kg IV- 2x/wk | TE maxillary first molars | 1 wk | 2 and 7 wk | 2 and 7 wk |
|  |  | Dexamethasone (Sigma, St. Louis) 5 mg/kg IV- 2x wk |  |  |  |  |

*NR = Not Reported; PD = Periapical disease; TE = Tooth Extraction; d = days; wk = weeks.

Symbol *indicate a statistically significant difference vs control at the same time point (p<0.05).
