# Supplementary figures and images for: Medication-related osteonecrosis of the jaws after tooth extraction in senescent female mice treated with zoledronic acid: Microtomographic, histological and immunohistochemical characterization
Source: PLoS One. 2019 Jun 14;14(6):e0214173. doi: 10.1371/journal.pone.0214173 (PMC6568384; doi:10.1371/journal.pone.0214173)

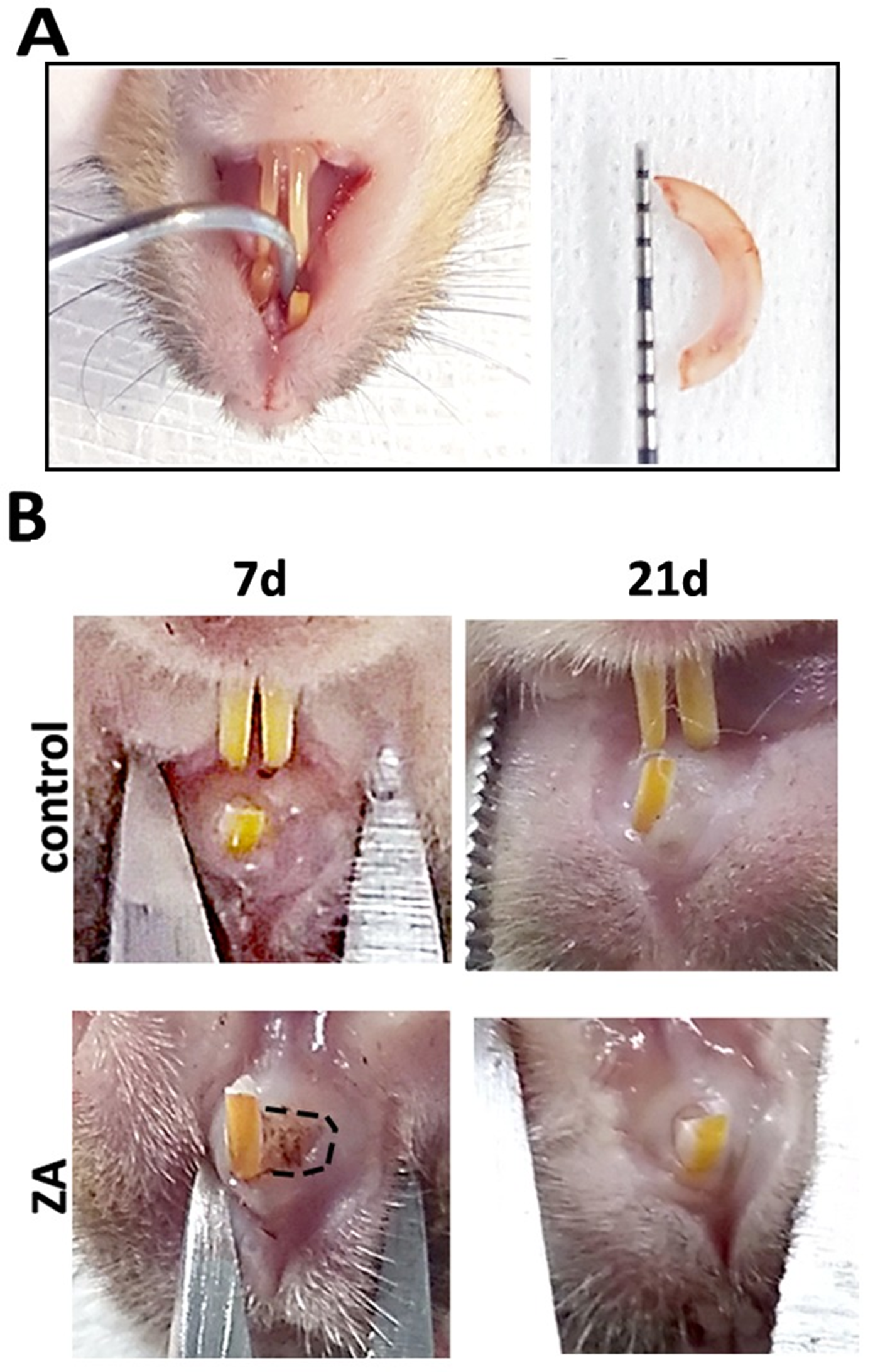

Supplement: S1 Fig — A) At 68 weeks of age, mice were subjected to atraumatically extraction of right upper incisor. B) Macroscopical occlusal views present clinical aspect of healing oral mucosal post-tooth extraction, 100% of control mice had a complete mucosal closure at 7d and 21 days, while 40% of ZA treated mice presented delayed in epithelial socket closure at 7 days (dotted line), but a complete mucosal closure at 21 days. (TIF) [file pone.0214173.s002.tif]

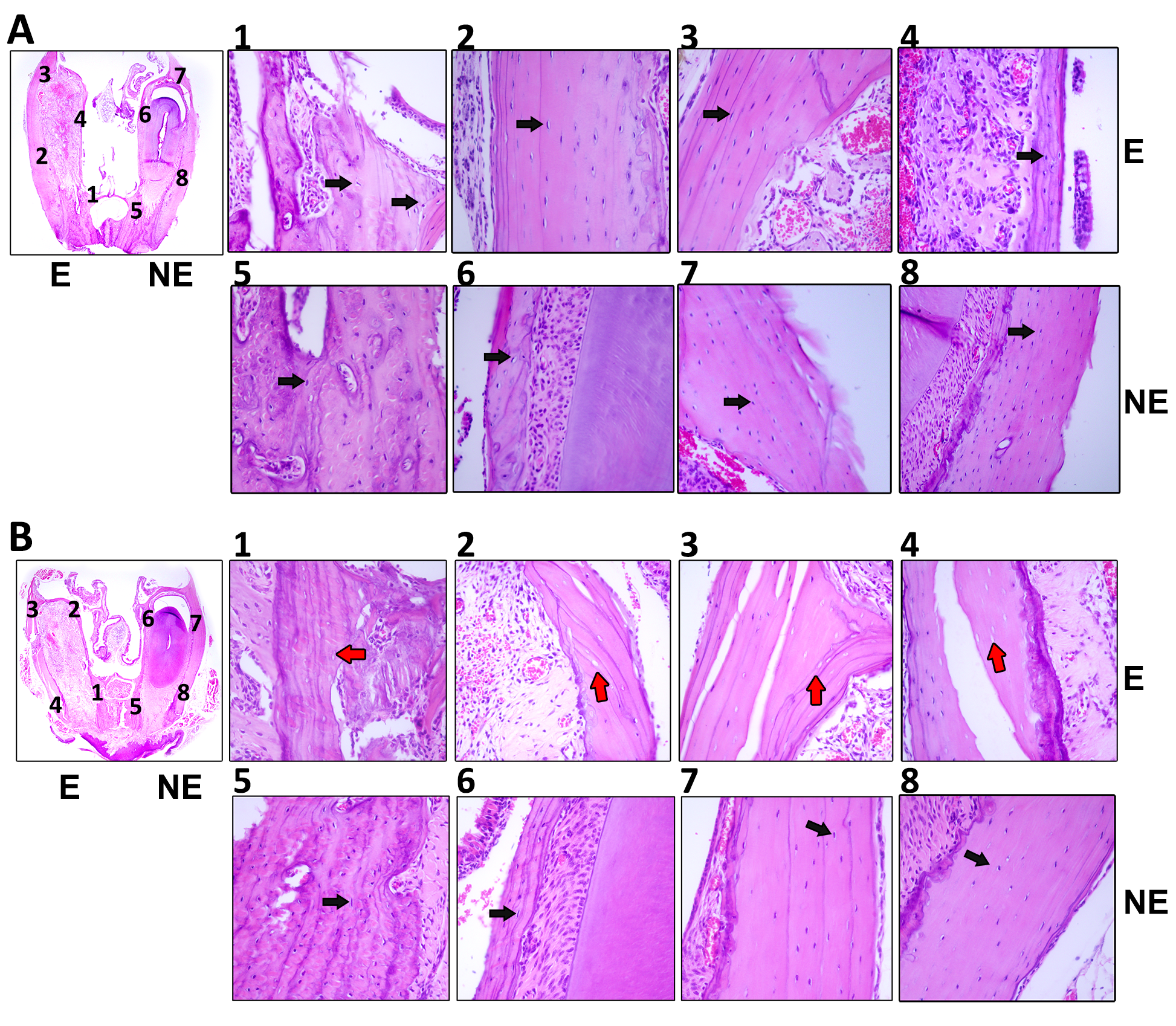

Supplement: S2 Fig — A) Control group extraction sites (A1-A4) and Control sites (A5-A8) filled with osteocytes (black arrows). B) ZA group extraction sites (A1-A4) present several empty lacunae (red arrows), while Control sites (A5-A8) remain filled with osteocytes (black arrows). Histological slides were stained with H&E (upper panel) and GT+Alcian blue (lower panel) and images were captured at 2x (entire section at left side) and 100x magnification (panels). (TIF) [file pone.0214173.s003.tif]

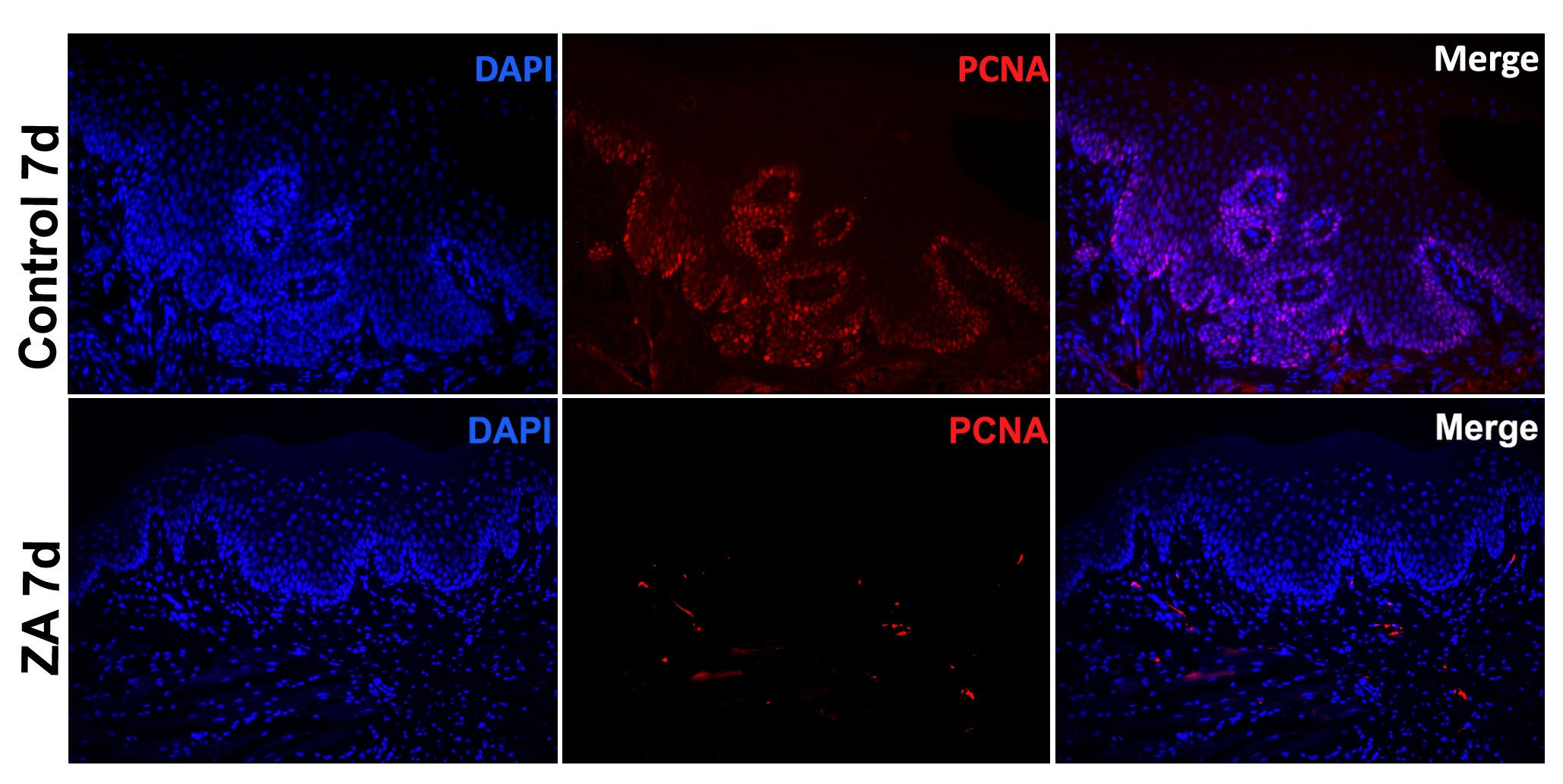

Supplement: S3 Fig — Mice received IP injections of 0.9% saline solution (Vehicle) or 250 μg/Kg one a week and upper right incisor were removed after 4 weeks of Vehicle or ZA treatments. Mice were euthanized for maxillary bones removal after 7days post tooth extraction. Secondary antibody Cy3 (#715-165-150, Jackson ImmunoResearch Laboratories, West Grove, PA, USA) for detection of PCNA and DAPI (D9542-50, Sigma-Aldrich Corp., St. Louis, MO, USA) for nuclear staining. (TIF) [file pone.0214173.s004.tif]
